# Chemical Composition and Phytotoxic Effects of Essential Oils of *Salvia hierosolymitana* Boiss. and *Salvia multicaulis* Vahl. var. *simplicifolia* Boiss. Growing Wild in Lebanon

**DOI:** 10.3390/molecules14114725

**Published:** 2009-11-19

**Authors:** Emilia Mancini, Nelly Apostolides Arnold, Laura De Martino, Vincenzo De Feo, Carmen Formisano, Daniela Rigano, Felice Senatore

**Affiliations:** 1 Dipartimento di Scienze Farmaceutiche, Università degli Studi di Salerno, Via Ponte Don Melillo, 84084 Fisciano, Salerno, Italy; E-Mails: emancini@unisa.it (E.M.); ldemartino@unisa.it (L.D.M.); 2 Faculté des Sciences Agronomiques, Université Saint Esprit, Kaslik (Beyrouth), Lebanon; E-Mail: arnoldnelly@hotmail.com (N.A.A.); 3 Dipartimento di Chimica delle Sostanze Naturali, Università degli Studi di Napoli “Federico II”, Via D. Montesano, 49, I-80131 Napoli, Italy; E-Mails: caformis@unina.it (C.F.); drigano@unina.it (D.R.); fesenato@unina.it (F.S.).

**Keywords:** *Salvia hierosolymitana* Boiss., *Salvia multicaulis* Vahl. var. *simplicifolia* Boiss., essential oil, germination, radical elongation

## Abstract

The chemical composition of the essential oils of *S. hierosolymitana* Boiss. and *S. multicaulis* Vahl. var. *simplicifolia* Boiss. collected in Lebanon was studied by means of GC and GC-MS analysis. In all 115 compounds were identified: 82 for S *hierosolymitana* and 72 for *S. multicaulis* var. *simplicifolia.* The presence of carbonylic compounds (17%) characterizes the oil from *S. hierosolymitana*, while S. *multicaulis* var. *simplicifolia* oil is rich of monoterpenes (34.5%) and sesquiterpenes (46.9%). The effects of the essential oils on germination and initial radical elongation of *Raphanus sativus* L. (radish) and *Lepidium sativum* L. (garden cress) were studied, indicating in a different activity against radical elongation of the species tested.

## 1. Introduction

Interactions between higher plants take place either by competition or by chemical inhibition or allelopathy. When the effect is due to the release of an effective phytotoxin, it is called allelopathy. Small quantities of toxins are responsible for massive reductions in plant growth. Allelopathy is one expression of the general phenomenon of chemical interaction and is probably of widespread significance in the functioning of natural communities. In fact, a number of plants have inhibitory effects on the growth of neighboring or successional plants by releasing allelopathic chemicals into the soil, either as exudates from living tissues or by decomposition of plant residues [1,2,3]. The study of compounds produced by plants, which inhibit or stimulate the germination and the development of other plants, is important for understanding the mechanisms of the ecological interaction. For this reason, our research group is carrying out a series of studies on the possible allelopathic properties of medicinal plants [4] that, being rich in active principles, are considered a primary source of potential allelochemicals. Aromatic species, in particular, are toxic almost without exception and the degree of toxicity seems to vary in effectiveness roughly with the intensity of aroma.

One of the best known and well-studied examples of allelopathy is the “*Salvia* phenomenon” [5]. The genus *Salvia* (Lamiaceae: subfamily Nepetoideae, tribe Mentheae) represents a cosmopolitan assemblage of nearly 1,000 species displaying a remarkable diversity in growth forms, secondary compounds, floral morphology and pollination biology. The first studies that demonstrated the presence of volatile growth inhibitors produced by *Salvia* species were carried out on *Salvia leucophylla* and *S. apiana* by Muller and co-workers [5]. The authors showed that when cucumber seedlings were placed in proximity to crushed leaves of the two *Salvia* species, their root growth was markedly inhibited, and this inhibition was increased as the amount of leaves was increased. Successively, the same authors suggested that the inhibition of growth of annual grassland species in and about colonies of *Salvia* and the gradually decreasing inhibition of herbs extending 9 m out into grassland from *Salvia* patches, were due to the production of volatile terpenes, particularly camphor and cineole [6,7,8,9].

In continuation of our studies on the possible phytotoxic activity of essential oils from plants collected in the Mediterranean area [4], we carried out *in vitro* experiments in order to verify the possible effects of the essential oils from *S. hierosolymitana* and *S. multicaulis* var. *simplicifolia* collected in Lebanon on germination and initial radical elongation of *Raphanus sativus* L. (radish) and *Lepidium sativum* L. (garden cress). 

*Salvia hierosolymitana* Boiss., Jerusalem sage, is also known by the Hebrew name *moriah* and the Arabic name *Quwaysah al quds, Lisân al’ijlah*. This plant is characterized by the presence of triterpenoids and shows antinflammatory [10] and antioxidant properties [11]. The essential oil was previously studied by us [12]. 

*Salvia multicaulis* Vahl. var. *simplicifolia* Boiss. is an evergreen shrub growing to 0.3 m × 0.25 m, native to South-West Asia, particular Eastern, Central, and Southern Turkey. Most of the components isolated from the extracts of *S. multicaulis* were terpenoids [13]. The essential oil of *S. multicaulis* from plants grown in Iran [14,15,16,17] and Lebanon [18] has been also previously reported. The essential oils and extracts of *S. multicaulis* collected in Turkey showed antimicrobial and antioxidative activities [19]. 

## Results and Discussion

The volatile components in the essential oils isolated from the two *Salvia* species and their percentage contribution are shown in Table 1, according to their elution order on a HP-5 MS column. The hydrodistillation yielded 0.32% and 0.13% of pale yellow oil (on a dry mass basis) for *S. hierosolymitana* and *S. multicaulis*. var. *simplicifolia*, respectively. One-hundred and fifteen compounds in all were identified, 82 for *S. hierosolymitana*, accounting for 86.6% of the total oil and 72 for *S. multicaulis* var. *simplicifolia* (90.8% of the oil). 

In *S. hierosolymitana* the monoterpenoid fraction amounted to 17.7% of the oil and was characterized only by oxygenated monoterpenoids, amongst which the most abundant was α-thujone (3.0%). The sesquiterpenoid fraction (16.5%) was mainly composed of sesquiterpenoid hydrocarbons (11.5%), with β-caryophyllene (2.4%) being the main compound. Spathulenol (1.9%) was the most abundant of the six oxygenated sesquiterpenoids identified. Noteworthy was the content of carbonylic compounds (17%), amongst which the C-18 ketone hexahydrofarnesyl acetone (5.3%) and the C-13 ketone β-ionone (3.5%) were particularly abundant. Also considerable was the phytol content (4.8%). Other components of the oil were fatty acids (21.4%) and hydrocarbons (3.7%), which were the most represented compounds, the former mostly represented by hexadecanoic acid (12.5%). 4-Vinyl-guaiacol (4.0%) was the sole phenolic compound determined.

In the oil from *S. multicaulis* var. *simplicifolia* the sesquiterpenoid fraction amounted to 46.9% of the total oil, while the monoterpenoid fraction was lower (34.5%). Differently from *S. hierosolymitana*, in which monoterpenoid hydrocarbons were completely absent, in *S. multicaulis* oil they accounted for the 10.6% and were particularly represented by α-pinene (5.5%). Also oxygenated monoterpenoids were abundant (23.9%) and in this fraction the main compounds were myrtenol (4.6%), sabinyl acetate (4.6%) and 1,8-cineole (3.1%). Among sesquiterpenoids, sesquiterpenoid hydrocarbons (38.6%) prevailed, particularly α-copaene (6.6%), β-caryophyllene (4.4%) and aromadendrene (3.9%). Another important difference in comparison with *S. hierosolymitana* essential oil was the paucity of carbonylic compounds (1.5%) and of fatty acids (1.2%). As stated before [18] the sample studied by us is different from the other Iranian samples studied by Ahmadi and Mirza [14] and Rustaiyan and co-workers [15]. Also the oils from Iran studied successively by Morteza-Semnani and co-workers [16] and Mohammadhosseini and co-workers [17] are different from our sample for the high content of camphor, 1,8-cineole and α-pinene that characterize them. The oil from Turkey presents the same features, being rich of α-pinene (21.9%), camphor (11%) and eucalyptol (20.1%) [19]. These differences might have been derived both from harvest time and local, climatic and seasonal factors or we may hypothesize that the Lebanese samples belong to a different chemotype than the Iranian and Turkish samples.

The oils from *S. hierosolymitana* and *S. multicaulis* var. *simplicifolia* from Lebanon were previously analyzed by us [12,18]; for the present study the aerial parts of the plants have been collected again in the same place (Kadiska valley and near Tannourine, both in Lebanon, respectively) and the GC and GC-MS analysis have been repeated on the new samples. As we can see (Table 1), data show that results obtained in the present study are similar to those of the previously studies [12,18], even if the percentages of some components are slightly different, thus confirming that the chemical composition of an essential oil depends strictly on the collection period.

The two essential oils were evaluated for their phytotoxic activity against germination and initial radical elongation of radish and garden cress, two species frequently utilized in biological assays [4]. The oils affected the germination and the radical elongation of radish and garden cress in a different way. Radical elongation seemed to be more affected than germination. The germination of radish did not appear significantly sensitive to the two essential oils (Table 2). Moreover, at a dose of 0.625 μg/mL the essential oil of *S. hierosolymitana* significantly inhibited the germination of radish. At the lowest dose tested the essential oil of *S*. *multicaulis* var. *simplicifolia* significantly promoted the germination of radish. The germination of garden cress was weakly inhibited in response to 0.125 μg/mL of essential oil of *S*. *multicaulis* var. *simplicifolia.* Radical elongation of radish was inhibited significantly in response to 0.125 μg/mL and 1.25 μg/mL of *S*. *multicaulis* var. *simplicifolia* (Table 3). Radical elongation of garden cress was promoted in response to 0.625 μg/mL of essential oil of *S. multicaulis* var. *simplicifolia* (Table 3). The difference in biological activity of the two oils could be attributed to their different chemical composition and to the presence, in *S. multicaulis* var. *simplicifolia* oil, of a major amount of oxygenated terpenoids, reported as germination and seedling growth inhibitors [20].

Our data agree with the literature on inhibitory activity exerted by essential oils of *Salvia* species on seed germination and radical elongation and in general on vegetation pattern. A dramatic example of zones free of annual herbs, influenced by terpenoids, was demonstrated by Muller [9] in California chaparral, in the areas surrounding patches of *Salvia leucophylla* L. (Labiatae) and *Artemisia californica* Lee. Volatile monoterpenoids emanating from leaves of *Salvia leucophylla* L. are responsible for anatomical and physiological changes occurring in herb seedlings which were exposed to vapors [21]. Camphor and 1,8-cineole, the main compounds of the oil of *Salvia leucophylla,* are potent inhibitors of oxygen uptake by mitochondrial suspensions [9].

Although the mode of inhibitory action of essential oils against germination still remains unclear, volatile oils and monoterpenoids inhibit cell division and induce structural breaks and decomposition in roots [22,23,24,25]. Both monoterpenoids and sesquiterpenoids appear to be involved in these allelopathic interactions. Several monoterpenoids are potent inhibitors of seed germination and seedling growth. These include 1,4 and 1,8-cineole [22], citronellal, citronellol, linalool [25,26], α-pinene [24,27], and limonene [27].

Recently, sesquiterpenoids (β-maaliene, α-isocomene, β-isocomene, δ-cadinene, 5-hydroxy-calamenene, and 5-methoxycalamenene) were shown to inhibit the seedling growth of associated native vegetation, and thus possibly help in successful invasion in the introduced sites [28]. 

## Experimental

### Plant material

Aerial parts of *S. hierosolymitana* Boiss were gathered at the full flowering stage from plants growing wild in the Kadiska valley (North Lebanon), on rocky soil, in June 2008, while aerial parts of *S. multicaulis* var. *simplicifolia* were collected near Tannourine (Lebanon), 1,700 a. s. l., in May 2008. Voucher specimens (leg. and det. N. Arnold s.n., confirm. Th. Raus) were deposited in the Herbarium of the Botanischer Garten, Berlin Universität, Germany.

### Isolation of the volatile components

Fifteen grams of each air-dried sample were ground in a Waring blender and then subjected to hydrodistillation for 3 hours according to the standard procedure described in the European Pharmacopoeia [29]. The oils were solubilised in *n*-hexane, filtered over anhydrous sodium sulphate and stored under N_2_ at +4 °C in the dark until tested and analyzed. 

### Gas chromatography

Analytical gas chromatography was carried out on a Perkin-Elmer Sigma-115 gas chromatograph equipped with a FID and a data handling processor. The separation was achieved using a HP-5 MS fused-silica capillary column (30 m × 0.25 mm i.d., 0.25 µm film thickness). Column temperature: 40 °C, with 5 min initial hold, and then to 270 °C at 2 °C/min, 260 °C (20 min); injection mode splitless (1 µL of a 1:1,000 *n*-pentane solution). Injector and detector temperatures were 250 °C and 290 °C, respectively. Analysis was also run by using a fused silica HP Innowax polyethylenglycol capillary column (50 m × 0.20 mm, 0.25 µm film thickness). In both cases, helium was used as carrier gas (1.0 mL/min).

### Gas chromatography–Mass spectrometry

Analysis was performed on an Agilent 6850 Ser. II apparatus, fitted with a fused silica DB-5 capillary column (30 m × 0.25 mm i.d.; 0.33 μm film thickness), coupled to an Agilent Mass Selective Detector MSD 5973; ionization energy voltage 70 eV; electron multiplier voltage energy 2000 V. Mass spectra were scanned in the range 40–500 amu, scan time 5 scans/s. Gas chromatographic conditions were as reported in the previous paragraph; transfer line temperature, 295 °C. 

### Identification of components

Most constituents were identified by gas chromatography by comparison of their Kovats retention indices (Ri) with either those of the literature [30,31] or with those of authentic compounds available in our laboratories. The Kovats retention indices were determined in relation to a homologous series of *n*-alkanes (C_8_-C_28_) under the same operating conditions. Further identification was made by comparison of their mass spectra on both columns with either those stored in NIST 02 and Wiley 275 libraries or with mass spectra from the literature [30,32] and a home made library. Components relative concentrations were obtained by peak area normalization. No response factors were calculated.

### Biological assay

A bioassay based on germination and subsequent radical growth was used to study phytotoxic effects of the essential oils of *S. hierosolymitana* and *S. multicaulis* var. *simplicifolia* on seeds of *Raphanus sativus* L. cv. “Saxa” (radish), and *Lepidium sativum* L. (cress). Seeds of *Lepidium sativum* L. and *Raphanus sativus* L. were purchased from Blumen srl, Piacenza, Italy. The seeds were surface-sterilized in 95% ethanol for 15 s and sown in Petri dishes (Ø = 90 mm), containing five layers of Whatman filter paper, impregnated with distilled water (7 mL, control) or tested solution of the essential oil (7 mL) at the different assayed doses. The germination conditions were 20 ± 1 °C, with natural photoperiod. The essential oils, in water–acetone mixture (99.5:0.5), were assayed at the doses of 2.5, 1.25, 0.625, 0.25, 0.125 and 0.062 μg/mL. Controls performed with water–acetone mixture alone showed no appreciable differences in comparison with controls in water alone. Seed germination was observed directly in Petri dishes, each 24 h. Seed was considered germinated when the protrusion of the radicle became evident [33]. After 120 hrs (on the fifth day), the effects on radical elongation were measured. Each determination was repeated three times, using Petri dishes containing 15 seeds each. Data are expressed as the mean ± SD of both germination and radical elongation [34]. The Student’s t test of independence was applied [35].

## Conclusions 

Aromatic plants are considered a primary source of potential allelochemicals and are toxic almost without exception. Previous studies show that *Salvia* species produce volatile growth inhibitors, particularly oxygenated monoterpenoids. Our *in vitro* experiments on the essential oils from *S. hierosolymitana* and *S. multicaulis* var. *simplicifolia* collected in Lebanon on germination and initial radical elongation of radish and garden cress, show that the essential oil of *S. multicaulis* var. *simplicifolia* was more active, whereas *S. hierosolymitana* oil didn’t show such activity. The phytotoxic activity of *S. multicaulis* var. *simplicifolia* is probably due to the presence of a substantial amount of oxygenated terpenoids, along with the presence of α-pinene (5.5%) and *p*-cymene (2.3%). Our *in vitro* studies can contribute to explain the importance of volatile compounds as chemical mediators in biochemical interactions among higher plants and can suggest models for lead compounds in the development of new pesticides [36].

## Figures and Tables

**Table 1:** Essential oil composition (%) of *Salvia hierosolymitana* (**H**) and *Salvia multicaulis* var. *simplicifolia* (**M**) growing wild in Lebanon.

R_i_^a^	R_i_^b^	Compound	Identification ^c^	H^d^	M^d^
**Monoterpenoid hydrocarbons**					**10.6**
938	1032	α-Pinene	1, 2, 3		5.5
953	1076	Camphene	1, 2, 3		0.9
973	1132	Sabinene	1, 2		0.4
980	1118	β-Pinene	1, 2, 3		0.9
993	1174	Myrcene	1, 2		0.3
1025	1280	*p*-Cymene	1, 2		2.3
1030	1203	Limonene	1, 2, 3		0.3
**Oxygenated monoterpenoids**				**17.7**	**23.9**
1034	1213	1,8-Cineole	1, 2, 3	0.8	3.1
1074	1482	*cis*-Linalool oxide (furanoid)	1, 2	0.4	
1085	1455	*trans*-Linalool oxide (furanoid)	1, 2	0.6	
1098	1553	Linalool	1, 2, 3	1.9	0.5
1105	1430	α-Thujone	1, 2	3.0	1.0
1115	1451	β-Thujone	1, 2	1.6	0.9
1137	1664	*trans*-Pinocarveol	1, 2		0.6
1145	1532	Camphor	1, 2, 3	0.9	0.8
1167	1719	Borneol	1, 2, 3		1.2
1176	1611	Terpinen-4-ol	1, 2, 3	0.5	1.2
1189	1706	α-Terpineol	1, 2	1.1	
1193	1648	Myrtenal	1, 2	0.7	1.8
1196	1804	Myrtenol	1, 2	0.6	4.6
1197	1597	Safranal	1, 2	0.4	
1217	1845	*trans*-Carveol	1, 2		0.2
1227	1698	Myrtenyl acetate	1, 2		1.8
1235	1857	Geraniol	1, 2	0.2	
1237	1665	Pulegone	1, 2	1.4	
1240	1656	Neral	1, 2	0.9	
1259	1665	Linalyl acetate	1, 2, 3	1.6	
1284	1597	Bornyl acetate	1, 2, 3		0.5
1295	1658	Sabinyl acetate	1, 2	0.6	4.6
1333	1709	α-Terpinyl acetate	1, 2		0.5
1343	1948	Piperitenone	1, 2	0.5	0.6
**Phenolic compounds**				**4.0**	**3.8**
1293	2198	Thymol	1, 2, 3		1.7
1299	2239	Carvacrol	1, 2, 3		0.6
1353	2186	Eugenol	1, 2, 3		1.5
1312	2180	4-Vinylguaiacol	1, 2	4.0	
**Sesquiterpenoid hydrocarbons**				**11.5**	**38.6**
1352	1466	α-Cubebene	1,2		0.3
1363	1492	Cyclosativene	1, 2	0.5	
1372	1493	Ylangene	1, 2		0.3
1377	1497	α-Copaene	1, 2	1.7	6.6
1385	1535	β-Bourbonene	1, 2	0.6	0.7
1387	1600	β-Elemene	1, 2		0.8
1411	1568	α-Cedrene	1, 2	0.5	
1415	1612	β-Caryophyllene	1, 2, 3	2.4	4.4
1437	1628	Aromadendrene	1, 2		3.9
1452	1673	(*E*)-β-Farnesene	1, 2	1.2	1.2
1455	1689	α-Humulene	1, 2	0.9	0.5
1463	1661	*allo*-Aromadendrene	1, 2	0.4	1.1
1475	*1715*	*β-Selinene*	1, 2	0.5	0.9
1477	1726	Germacrene D	1, 2		0.6
1478	1704	γ-Muurolene	1, 2	0.4	2.0
1483	*1784*	*ar*-Curcumene	1, 2		1.1
1491	1695	Viridiflorene	1, 2		0.9
1495	1740	Valencene	1, 2	t	1.2
1498	1744	α-Selinene	1, 2	0.5	0.4
1500	1740	α-Muurolene	1, 2	0.2	1.3
1505	1758	(*E,E*)-α-Farnesene	1, 2		0.6
1515	1776	γ-Cadinene	1, 2	1.2	2.9
1520	1839	1S-*cis*-Calamenene	1, 2	0.3	1.5
1526	1773	δ-Cadinene	1, 2	0.1	3.0
1541	1941	α-Calacorene	1, 2	t	0.9
1677	2256	Cadalene	1, 2	0.1	1.5
**Oxygenated sesquiterpenoids**				**5.0**	**8.3**
1565	2057	Ledol	1, 2	0.6	0.6
1566	2050	(*E*)-Nerolidol	1, 2		1.4
1578	2150	Spathulenol	1, 2	1.9	0.7
1580	2008	Caryophyllene oxide	1, 2, 3		0.8
1585	2098	Globulol	1, 2	0.2	0.4
1591	2104	Viridiflorol	1, 2		0.9
1605	2011	Humulene epoxide II	1, 2		0.5
1629	2025	*epi*-Globulol	1, 2		0.6
1640	2187	T-Cadinol	1, 2	1.2	
1642	2209	T-Muurolol	1, 2	0.6	0.5
1644	2080	Cubenol	1, 2		0.6
1645	2145	Torreyol	1, 2		0.1
1649	2255	α-Cadinol	1, 2	0.5	0.8
1650	2257	β-Eudesmol	1, 2		0.4
1780	2478	14-Hydroxy-α-humulene	1, 2		t
**Carbonylic compounds**				**17.0**	**1.5**
901	1238	(*Z*)-4-Heptenal	1, 2	t	
963	1543	Benzaldehyde	1, 2, 3	0.7	
975	1312	1-Octen-3-one	1, 2	0.1	
1015	1507	(*E,E*)-2,4-Heptadienal	1, 2	0.2	
1048	1663	Phenylacetaldehyde	1, 2, 3	1.0	
1055	1466	(*E*)-2-Octenal	1, 2	0.5	
1154	1572	(*E,Z*)-2,6-Nonadienal	1, 2	t	
1184	1797	*p*-Methylacetophenone	1, 2	0.5	
1206	1510	Decanal	1, 2	t	
1382	1838	(*E*)-β-Damascenone	1, 2	2.2	0.2
1454	1854	(*E*)-Geranyl acetone	1, 2	0.8	
1482	1957	(*E*)-β-Ionone	1, 2, 3	3.5	
1580	1815	Tridecan-2-one	1, 2	0.3	
1694	2031	*Pentadecan-2-one*	1, 2	0.7	
1835	2131	Hexahydrofarnesyl acetone	1, 2	5.3	1.3
1918	2384	(*E,E*)-Farnesyl acetone	1, 2	1.2	
**Hydrocarbons**				**3.7**	**2.3**
1179	1763	Naphtalene	1, 2, 3	0.3	
1208	1567	α-Ionene	1, 2	0.3	
2400	2400	Tetracosane	1, 2, 3	0.1	
2500	2500	Pentacosane	1, 2, 3	0.5	0.7
2600	2600	Hexacosane	1, 2, 3	0.2	
2700	2700	Heptacosane	1, 2, 3	1.0	0.7
2800	2800	Octacosane	1, 2, 3	t	
2900	2900	Nonacosane	1, 2	0.8	0.9
3000	3000	Triacontane	1, 2, 3	t	
3100	3100	Entriacontane	1, 2, 3	0.5	
3200	3200	Dotriacontane	1, 2, 3	t	
**Fatty acids**				**21.4**	**1.2**
1957	2931	Palmitic acid	1, 2, 3	12.5	1.2
1568	2467	Dodecanoic acid	1, 2, 3	0.1	
1768	2672	Tetradecanoic acid	1, 2, 3	1.5	
1873	2740	Pentadecanoic acid	1, 2, 3	0.2	
2099	3195	(*Z,Z,Z*)-9,12,15-Octadecatrienoic acid	1, 2, 3	2.2	t
2104	3160	(*Z,Z*),9,12-Octadecadienoic acid	1, 2, 3	3.3	
2120	3157	(*Z*)-9-Octadecenoic acid	1, 2, 3	1.6	
**Others**				**7.0**	**0.6**
1002	1243	2-Pentylfuran	1, 2	t	
1290	2471	Indole	1, 2, 3	0.7	
1485	2354	Dihydroactinidiolide	1, 2	1.3	
1672	2175	Tetradecanol	1, 2	0.2	
1950	2622	Phytol	1, 2	4.8	0.6

^a^: Kovats retention index on HP-5 MS column; ^b^: Kovats retention index on HP Innowax; ^c^: 1 = Kovats retention index, 2 = mass spectrum, 3 = coinjection with authentic compound; ^d^: t = trace, less than 0.05%.

**Table 2:** Biological activities of essential oils of *Salvia hierosolymitana* and *S. multicaulis* var. *simplicifolia* against germination of *Raphanus sativus* (radish) and *Lepidium sativum* (garden cress), 120 hrs after sowing. Results are shown as mean ± standard deviation (SD) of three experiments.

	*Salvia hierosolymitana*	*Salvia multicaulis* var. *simplicifolia*
***Raphanus sativus***	**Germinated seeds ± SD**	**Germinated seeds ± SD**
**Control**	12.67 ± 1.51	10.67 ± 1.37
**0.062** μg/mL	13.33 ± 1.15	13.00 ± 1.00 ***
**0.125** μg/mL	13.33 ± 0.58	11.00 ± 2.00
**0.250** μg/mL	13.67 ± 0.58	12.33 ± 1.53
**0.625** μg/mL	8.33 ± 2.31*	12.67 ± 2.52
**1.25** μg/mL	11.67 ± 1.53	12.00 ± 1.00
**2.50** μg/mL	12.00 ± 0.00	11.33 ± 1.53
***Lepidium sativum***	**Germinated seeds ± SD**	**Germinated seeds ± SD**
**Control**	11.17 ± 2.04	11.17 ± 1.47
**0.062** μg/mL	11.33 ± 1.15	11.33 ± 2.08
**0.125** μg/mL	11.67 ± 4.16	8.33 ± 2.08 *
**0.250** μg/mL	12.00 ± 2.00	10.33 ± 2.52
**0.625** μg/mL	12.00 ± 1.73	12.00 ± 2.65
**1.25** μg/mL	11.67 ± 2.08	12.00 ± 1.73
**2.50** μg/mL	12.33 ± 0.58	10.67 ± 0.58

Note: *p < 0.05; **p < 0.01; ***p < 0.001 vs positive control.

**Table 3:** Biological activities of essential oils of *Salvia hierosolymitana* and *S. multicaulis* var. *simplicifolia* against radical elongation of *Raphanus sativus* (radish) and *Lepidium sativum* (garden cress), 120 hrs after sowing. Data are expressed in cm. Results are shown as mean±standard deviation (SD) of three experiments.

	*Salvia hierosolymitana*	*Salvia multicaulis* var. *simplicifolia*
***Raphanus sativus***	**Radicle length ± SD (cm)**	**Radicle length ± SD (cm)**
**Control**	3.14 ± 2.24	3.89 ± 2.39
**0.062** μg/mL	2.60 ± 1.59	3.34 ± 2.03
**0.125** μg/mL	2.80 ± 2.01	2.62 ± 1.77***
**0.250** μg/mL	3.37 ± 2.00	3.18 ± 1.31
**0.625** μg/mL	2.71 ± 1.81	4.20 ± 2.62
**1.25** μg/mL	3.04 ± 1.80	2.13 ± 1.97***
**2.50** μg/mL	2.80 ± 2.07	3.29 ± 2.02
***Lepidium sativum***	**Radicle length ± SD (cm)**	**Radicle length ± SD (cm)**
**Control**	3.47 ± 1.78	3.21 ± 1.60
**0.062** μg/mL	3.08 ± 1.58	2.64 ± 1.67
**0.125** μg/mL	3.31 ± 2.36	4.05 ± 2.51
**0.250** μg/mL	3.19 ± 1.67	3.61 ± 1.63
**0.625** μg/mL	3.25 ± 1.80	4.12 ± 1.34**
**1.25** μg/mL	3.18 ± 1.60	3.83 ± 1.85
**2.50** μg/mL	2.99 ± 1.51	2.97 ± 1.82

Note: *p < 0.05; **p < 0.01; ***p < 0.001 vs positive control.
